# Micro-CT evaluation of dentinal microcrack formation in mesiobuccal canals of maxillary molars following instrumentation with heat-treated rotary and reciprocating systems

**DOI:** 10.1186/s12903-025-05929-z

**Published:** 2025-04-11

**Authors:** Fatemeh Soltaninejad, Yazdan Shantiaee, Babak Zandi, Arsham Moslemi, Seyed Sepehr Mirebeigi-Jamasbi

**Affiliations:** 1 Department of Endodontics, School of Dentistry, Shahid Beheshti University of Medical Sciences, Tehran, Iran; 2 Research Committee, School of Dentistry, Shahid Beheshti University of Medical Sciences, Daneshju Blvd., Velenjak St., Chamran Highway, Tehran, 1983963113 Iran

**Keywords:** Dentinal microcrack, Micro-CT, Root Canal Preparation, Rotary file systems, Reciprocating file systems

## Abstract

**Background:**

The goal of root canal preparation is to eliminate microorganisms and debris from the root canal system, while providing space for the placement of root filling materials. However, the process can lead to the formation of dentin microcracks, increasing the risk of vertical root fractures. This study used micro-CT to evaluate the formation of dentinal microcracks following instrumentation with One Curve, EdgeOne Fire, and VDW.ROTATE systems.

**Methods:**

Thirty-three extracted maxillary molars with non-calcified Vertucci type 4 s mesiobuccal canals were included. After pre-instrumentation micro-CT scans, a single endodontist prepared the canals with three aforementioned rotary systems. Post-instrumentation scans were conducted and axial sections from both the pre- and post-preparation scans were analyzed by two blinded observers to identify newly formed microcracks. Cohen’s Kappa coefficient assessed interobserver agreement and dentinal microcracks were evaluated using ANOVA, ANCOVA, Kruskal-Wallis, and McNemar’s test (*P* < 0.05).

**Results:**

All three rotary systems led to significant increases in dentinal microcracks post-instrumentation. While the VDW.ROTATE group showed a slightly higher percentage of cracked Sect. (72%), statistical analysis showed no significant difference between the groups (*P* = 0.35).

**Conclusion:**

Preparation of maxillary molar root canals using different rotary systems resulted in the formation of dentin microcracks, with no significant correlation to the specific system used.

## Background

The primary objective of chemo-mechanical root canal preparation is to eliminate microorganisms, pulp tissue, and debris from the root canal system while shaping the canal to provide adequate space for the placement of root filling materials. Advances in nickel-titanium (NiTi) instruments have greatly facilitated this process [1, 2]. Various manufacturers have enhanced these tools by improving key features such as flexibility and efficiency to reduce treatment time. Rotary systems for root canal preparation can be categorized into two types. The first is the multiple-file (sequential) method, where several files are used in a specific sequence to shape the canal, as seen with VDW.ROTATE system. The second method involves single-file systems, such as One Curve, which rely on a single file during preparation and are favored for their simplicity and ease of use [3].

The One Curve system is a single-file, heat-treated NiTi instrument introduced in 2018, made from C-Wire alloy. This design leverages shape memory technology, allowing the file to maintain flexibility while effectively shaping canals. It features a size 25 tip, a constant 6% taper, and variable cross-sections, transitioning from a triangular shape at the tip to an S-shaped configuration near the shaft, which is intended to optimize cutting efficiency and maintain a centered trajectory [4].

The VDW.ROTATE system, made from heat-treated Blue-wire NiTi alloy, offers a range of taper sizes tailored for narrow root canals [5]. Its design includes a coronal taper to preserve dentin and facilitate preparation in canals with limited access. The files (sizes 15/0.04, 20/0.05, and 25/0.04) feature an S-shaped cross-section, an off-centered design, and a constant taper, enhancing cutting performance and reducing canal wall stress [6].

EdgeOne Fire, a reciprocating file system constructed from FireWire NiTi alloy, is available in 21 mm, 25 mm, and 31 mm lengths. It is claimed to have five times the cyclic fatigue resistance of WaveOne Gold while being cheaper. EdgeOne Fire operates with the same reciprocating angles as WaveOne Gold, allowing users to switch easily between the two systems [7].

During root canal preparation, an amount of dentin is removed from the canal wall, which can increase the tooth’s susceptibility to root fractures [8]. Research has indicated that the process of root canal preparation may contribute to the formation of dentin microcracks within the canal walls [9]. Under masticatory and physiological forces, these microcracks can propagate and evolve into VRFs, potentially resulting in tooth loss [10]. Improper matching of the file to the canal’s anatomy can lead to uneven dentin removal, unnecessarily weakening the root structure [11]. Microscopic studies have demonstrated a correlation between root canal preparation and the formation of microcracks [10]. These microcracks are thought to arise due to lateral forces and strain exerted on the canal walls, which is particularly evident when using instruments with larger tapers or when treating teeth with significant root curvature [12].

Some studies using tooth sectioning followed by light microscopy have identified a relationship between root canal preparation with rotary instruments and the formation of microcracks in the canal walls [13, 14]. However, modern approaches recommend the use of micro-computed tomography (micro-CT) as it is a non-destructive technique, preserving tooth integrity while allowing detailed evaluation of dentin and its alterations. Due to its high precision, micro-CT is now widely used for assessing dentin microcracks following the use of rotary files [9, 15]. Discrepancies exist among the findings of various studies. For instance, Cassemiro et al., De-Deus et al., and Shantiaee et al. reported no dentin microcracks resulting from any of the preparation methods investigated, whereas Ceyhanli et al. found that the rotary files used in their study increased the incidence of microcracks [9, 15–17]. To the best of our knowledge, no previous study has evaluated microcrack formation associated with the VDW.ROTATE system or directly compared it with the One Curve and EdgeOne Fire systems.

The aim of the present study is to use micro-CT to assess the formation of new microcracks following the preparation of the second mesiobuccal canal of maxillary molars using One Curve, EdgeOne Fire, and VDW.Rotate file systems. The study includes two null hypotheses. The first one states that there is no significant dentinal microcrack formation when using the One Curve, EdgeOne Fire, and VDW.Rotate file systems. The second null hypothesis states that there is no difference in the amount of microcrack formation among these file systems.

## Methods

This ex vivo study was conducted following approval by the Ethics Committee of Shahid Beheshti University of Medical Sciences (IR.SBMU.DRC.REC.1401.071). Human maxillary molars, previously extracted due to periodontal problems at the Oral and Maxillofacial Surgery Department of Shahid Beheshti Dental School, were collected from 10 January 2023 to 20 January 2023 and used for the investigation. The teeth were anonymized, and the authors did not have access to any information that could identify individual participants during or after data collection. Teeth were included if they had mesiobuccal roots with a second mesiobuccal canal classified as Vertucci type 4, with a length of 19 to 22 mm, and a canal curvature between 20 and 40 degrees. A stereomicroscope (Zeiss Stemi 305, Oberkochen, Germany) was utilized for the initial evaluation of the teeth, similar to previous studies [15, 18, 19]. The canal had to be non-calcified and able to pass a size 8 file. Teeth with immature apices, external or internal resorption, calcification, root decay, cracks, as well as those with previous root canal treatments, were excluded from the study. Based on an α of 0.05 and a power of 80%, the sample size for each group was calculated to be 11 teeth, with a total of 33 teeth included in the study. To select eligible teeth, candidate teeth stored in a 0.1% thymol solution were first prepared by separating the crowns from the roots using a diamond disc under water cooling. The roots were then examined for cracks under x12 magnification using a stereomicroscope. A size 8 K-file (Mani Inc., Tochigi, Japan) was used to determine both the working length and Vertucci canal type. To provide an artificial periodontal ligament space, similar to previous studies, a single layer of aluminum foil was wrapped around the roots before embedding them in tubes filled with acrylic resin (Kulzer, Hanau, Germany). Once the resin had set, the aluminum foil was removed, creating a space around the root. This space was then filled with silicone imoression material (GC Corporation, Tokyo, Japan), and the root was promptly repositioned in the block [17, 19]. One week prior to root canal preparation, the presence of calcification was assessed by analyzing axial sections obtained from the LOTUS inVivo device (Behin Negareh Co., Tehran, Iran), using exposure settings of 80 kV, 40 µA, with a 2-second exposure time and a resolution of 16 micrometers.

The eligible teeth were randomly assigned to the three preparation system groups, with 11 teeth in each group. In the One Curve group (Micro Mega, Besancon, France), a single-file system, after confirming canal patency with a size 8 K-file (Mani Inc., Tochigi, Japan), the One G glider file was used with continuous rotation at a speed of 250–400 rpm and a torque of 1.2 N·cm, using the X-Smart rotary motor (Dentsply Sirona, Ballaigues, Switzerland). Once the glider file reached the working length, the main file (size 25/0.06) was employed at a speed of 300–450 rpm and a torque of 2 N·cm. Each time the file entered the root canal, it was accompanied by a brushing motion along the canal walls. After three strokes, the file was removed, the debris was cleared using sterile gauze, and the canal was rinsed with 10 mL of normal saline. The file was then reinserted into the canal until it reached the working length. This process was followed for all canals. In the VDW.ROTATE group (VDW, Munich, Germany), a sequential rotary system, the same protocol as the One Curve group was used. A 15/0.04 glide path file was employed with a torque of 1.3 N·cm and a speed of 350 rpm using the X-Smart rotary motor. The enlargement file (size 20/0.05) and the main file (25/0.06) was used with a speed of 350 rpm and a torque of 2 N·cm. In the EdgeOne Fire group (Edge Endo, Albuquerque, New Mexico, USA), reverse reciprocating movements were used. The EdgeOne Fire Glide Path file acted as the glider file, and the main file was size 25/0.06. The X-Smart rotary motor, set to the WaveOne mode, was used with a speed of 350 rpm and reciprocal movements of 150 degrees counterclockwise and 30 degrees clockwise.

**Fig. 1:**
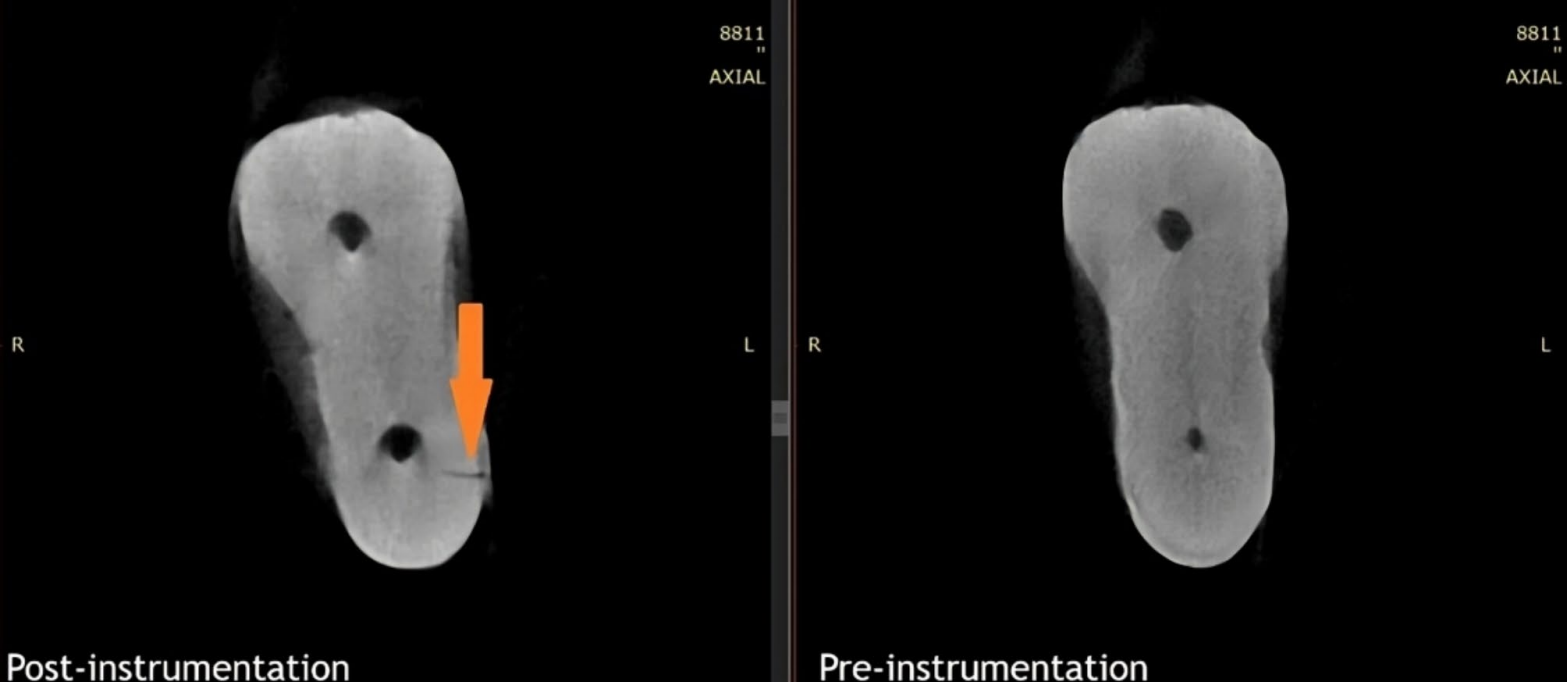
Evaluation of axial micro-CT sections before and after preparation to find microcracks. The arrow indicates a microcrack in this sample

The canal preparation procedures were performed by an endodontist at the same time each day, with a maximum of five canals prepared daily. This approach was taken to ensure consistent application of force during preparation and to minimize the potential impact of operator fatigue on the results. Additionally, to prevent file fatigue or structural changes, each file was used for the preparation of three canals. After completing the preparation, the samples were rescanned using micro-CT with the same parameters as before preparation. Axial sections from both the pre- and post-preparation scans were examined simultaneously, similar to many previous studies assessing dentinal microcracks [9, 15–17]. Two blinded observers reviewed all sections and recorded those showing microcracks (Fig. 1).

**Fig. 2:**
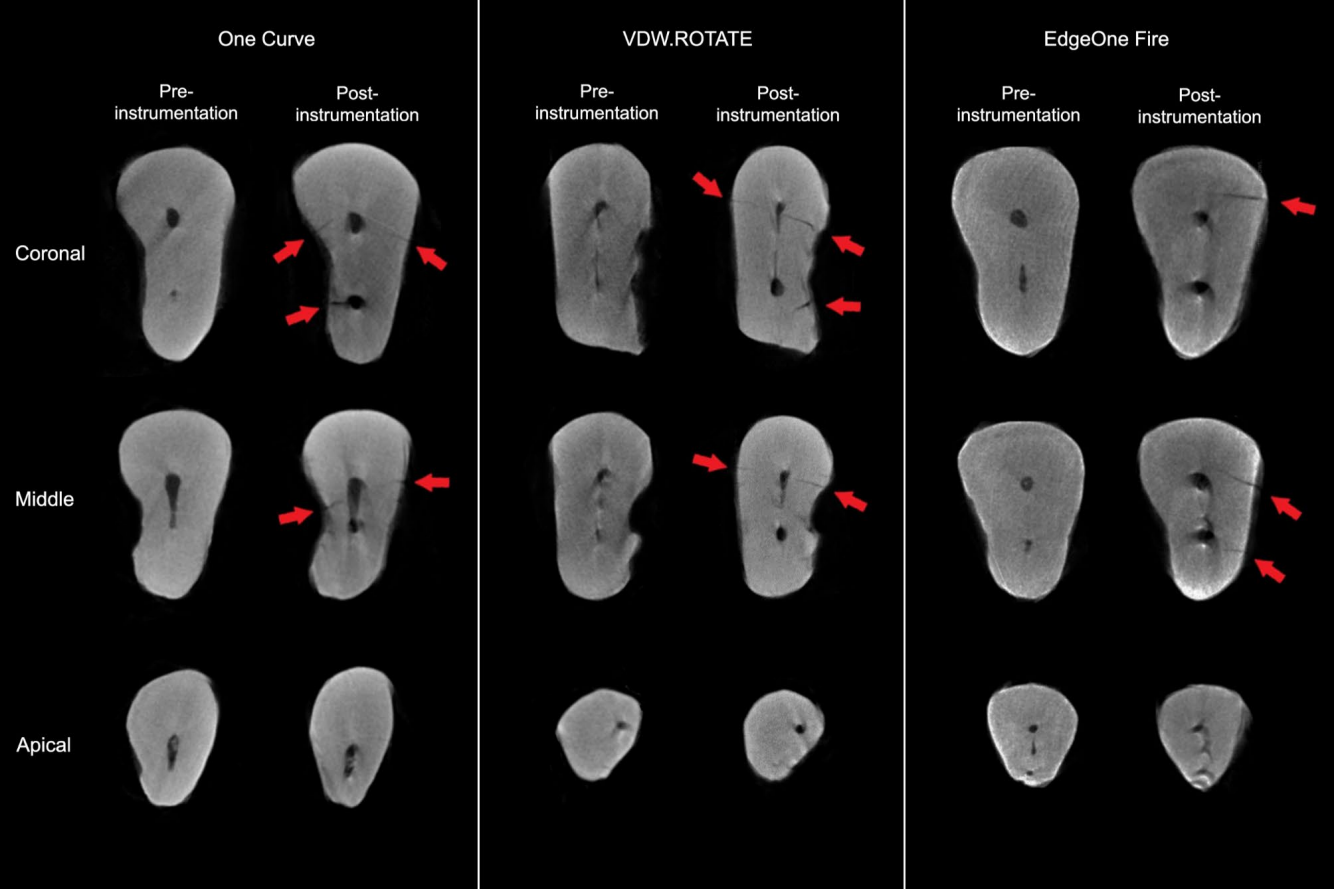
Examples of micro-CT sections before and after root canal preparation for each study group. Arrows indicate the presence of microcracks

Cohen’s Kappa coefficient was calculated to assess the level of agreement between the observers. The McNemar test was applied to compare the presence of microcracks before and after preparation within each group. ANCOVA was used to compare the number of sections with dentin microcracks after preparation across the three groups, accounting for differences in the number of microcracked sections before preparation. The ANOVA test was used to compare the number of newly formed dentin microcracks among the groups, while the Kruskal–Wallis test was employed to compare the number of microcracked sections among the three groups before preparation. We considered *P* < 0.05 as statistically significant.

## Results

The Kappa coefficient between the two observers was 0.72, indicating substantial agreement. Out of a total of 21,674 micro-CT axial sections analyzed before and after preparation, 7,656 Sect. (35.3%) showed dentin microcracks. In the pre-preparation images, 353 Sect. (3.2%) exhibited microcracks, which increased to 7,303 Sect. (67.3%) after the preparation. In the first-stage scans, the number of microcracks in the One Curve, VDW.ROTATE, and EdgeOne Fire groups were 0 (0%), 13 (0.3%), and 340 (9.4%), respectively. In the second-stage scans, the number of microcracked sections rose to 2,468 (67.2%) in the One Curve group, 2,586 (72%) in the VDW.ROTATE group, and 2,249 (62.8%) in the EdgeOne Fire group (Fig. 2). The increase in microcracks within each group was statistically significant (Table 1).

**Table 1:** The number of sections with microcracks in each group before and after Preparation

Study groups	Stage	Total sections	Sections with microcracks	*P* value
**One Curve**	Before preparation	3669	0 (0%)	< 0.001
	After preparation	3669	2468 (67.2%)	
**VDW.ROTATE**	Before preparation	3588	13 (0.3%)	< 0.001
	After preparation	3588	2586 (72%)	
**EdgeOne Fire**	Before preparation	3580	340 (9.4%)	< 0.001
	After preparation	3580	2249 (62.8%)	

The number of sections with microcracks before preparation was significantly higher in the EdgeOne Fire group compared to the other two groups (*P* = 0.003). As a result, ANCOVA was used to compare the number of sections containing microcracks after preparation, accounting for the initial difference among the groups. The analysis showed no significant difference in the final number of cracks between the groups (*P* = 0.35). Additionally, the increase in newly formed microcracks was compared between the groups and the difference was not statistically significant (*P* = 0.175).

## Discussion

Root canal preparation results in the removal of some root dentin, consequently weakening the tooth structure. Research indicates that dental cracks occur when the tensile stress on the root canal walls exceeds the tensile strength of the dentin [20]. These microcracks can propagate under physiological and pathological occlusal forces, during root canal retreatment, post space preparation, or post placement. Over time, they may develop into complete cracks or vertical root fractures, potentially leading to tooth extraction [8]. This study aimed to assess the formation of dentin microcracks following canal preparation using One Curve, EdgeOne Fire, and VDW.ROTATE preparation systems, employing micro-CT, which is currently the most precise method for evaluating dentin microcracks [21]. All three systems led to a statistically significant formation of dentin microcracks. However, the differences between the groups were not statistically significant.

In a recent study, the single-file One Curve system generated fewer dentinal cracks compared to the multi-file ProTaper Next system, even under varying torque settings [4]. Sindi et al. [22] compared Reciproc, One Curve, and Vortex Blue systems, reporting that while all systems caused some microcracks, the differences were not statistically significant, aligning with the results of this study. Similarly, a 2021 investigation involving Edge Taper Platinum, ProTaper Gold, Reciproc Blue, and EdgeOne Fire systems also found no significant differences among the groups regarding crack formation [23]. To the best of our knowledge, the present study is the first to evaluate microcrack formation associated with the VDW.ROTATE system. Additionally, no prior research has directly compared dentinal crack formation between the One Curve and EdgeOne Fire systems.

Previous studies have reported varying results, which may be partly due to differences in the methods used to investigate microcracks. Some studies relied on sectioning and examination under a stereomicroscope [9, 24]. The limitation of this approach is that it does not allow for the evaluation of sections prior to root canal preparation, making it difficult to distinguish between pre-existing and newly formed microcracks, which can introduce errors in the analysis. One suggested factor contributing to the presence of microcracks before preparation is the extraction process itself, as it exerts force on the tooth structure [25]. Additionally, dehydration of samples during testing can cause dentin shrinkage, potentially leading to crack formation and reduced fracture resistance [26, 27]. The sectioning process may also introduce stress, leading to the creation of additional microcracks.

In this study, we employed the Micro-CT method, which is not only highly accurate but also minimally invasive, allowing for three-dimensional assessment of the entire root canal. The findings of this study differ from many previous reports that have shown no formation of new microcracks after preparation [15–19, 28, 29]. However, some studies using Micro-CT have reported the formation of dentin microcracks, consistent with the results of the present study [9, 30, 31].

Several factors may account for the discrepancies between our findings and previous studies. It has been reported that greater root canal curvature is associated with an increased risk of dentinal microcrack formation. In the present study, the root curvatures ranged from 20 to 40 degrees, similar to the study by Ceyhanli et al. [9], who reported findings consistent with ours. In contrast, studies that found no significant effect of preparation on microcrack formation, such as those by De-Deus et al. (10–20 degrees), Bayram et al. (< 5 degrees), and Cassimiro et al. (< 5 degrees), utilized samples with lower curvatures [15, 16, 18]. Additionally, the rotational movement of instruments with higher torque can cause higher stress distribution on dentin, contributing to greater microcrack formation [9]. In the present study, a torque of 2 N·cm was applied during rotational movements, which is higher than the 1.5 N·cm torque used in the study by Shantiaee et al. [17]. Moreover, high rotational speeds have been suggested to enhance cutting efficiency and reduce defect formation [32]. While we used a rotational speed of approximately 350 rpm, Bayram et al. [18] employed a higher speed of 500 rpm for root canal preparation.

Another important consideration is the variability in the resolution of Micro-CT imaging across studies, which has ranged from 6.8 μm to 70 μm. Differences in resolution can impact the accuracy of detecting dentinal microcracks, potentially explaining the conflicting results among studies. Furthermore, anatomical variations in root canal morphology across different studies may have influenced the findings. Since canal anatomy affects the degree of friction between the instrument and the canal wall, differences in morphology may lead to varying stress distributions, ultimately influencing microcrack formation.

Other factors, such as storage conditions and the age of the teeth, may have also influenced the results. Prior studies have highlighted that these variables can affect dentinal defect formation [33, 34]. However, many of the aforementioned studies did not provide sufficient details regarding the age of the tooth donors or the storage conditions following extraction. This lack of standardization could have impacted the results. Notably, our study was conducted on teeth extracted due to periodontal disease, making it highly likely that they originated from older individuals. Given that aging alters dentin properties, such as its resistance to crack propagation [34], this factor may have contributed to the observed differences. These findings emphasize the need for future studies to use standardized tooth samples, extracted from individuals within a specific age range, and stored under controlled conditions to ensure better comparability across studies.

In terms of file motion, the EdgeOne Fire system, which utilizes reciprocating motion, produced fewer microcracks compared to the two rotary systems. Unlike rotary systems that continuously apply stress to the canal walls during preparation, reciprocating systems apply intermittent stress. Reciprocating motion has also been shown to maintain the canal’s center more effectively [35]. Furthermore, with this motion, the file is automatically released from the canal by a clockwise movement, reducing bending and torsional stress on the canal walls, which may help prevent microcrack formation [36]. Many studies have indicated that reciprocating systems cause fewer dentin microcracks compared to rotary systems [37–39]. However, some studies have found no difference between the two motions in microcrack formation [24, 40]. Conversely, Koroth et al. and Haridas et al. concluded that reciprocating movements led to more dentin microcracks than rotary movements [41, 42]. These conflicting results could be due to other factors, such as file design and additional variables influencing microcrack formation. It is established that files with greater taper exert more pressure on canal walls [43]. Thompson suggested that several factors, including tip design, cross-sectional geometry, taper, and flute shape of rotary instruments, could influence crack formation [44]. In the present study, the final file used in all three groups had a 6% taper. Additionally, crown separation was performed before the first-stage Micro-CT scan to isolate the effects of canal preparation and eliminate any impact from access cavity preparation on the dentin. By removing the crown, we also eliminated crown-related variables such as anatomy and access type, allowing for a more accurate comparison of the preparation methods.

While micro-CT is considered the gold standard for examining dentin microcracks, it does have its limitations. Variations in exposure parameters, resolution, and data reconstruction make it difficult to directly compare results of different studies [18, 29]. The ability to detect dentin defects is heavily influenced by the isotropic resolution of the Micro-CT scan [45]. In this study, an isotropic voxel size of 16 μm was used, meaning microcracks smaller than 16 μm might not have been detected. Another limitation of micro-CT is the presence of image reconstruction artifacts, which can be mistaken for microcracks or may obscure existing cracks, complicating their detection. However, performing sequential analyses of cross-sections throughout the root helps to differentiate microcracks from artifacts [46]. The moisture level of the sample during the micro-CT scanning process also poses challenges. Rodig et al. found that detecting dentin microcracks in overly moist alveolar bone blocks is difficult. Excessive moisture in dentin may result in poor visibility of microcracks in micro-CT images, potentially causing false negatives [47]. In this study, samples were dried for at least 24 h before micro-CT scanning, improving the detection of microcracks. Nevertheless, the ideal moisture conditions seem to lie somewhere between ensuring easy visibility of microcracks and preventing the formation of new defects due to dehydration [26, 46]. Identifying the optimal moisture balance for micro-CT imaging remains a significant challenge when examining dentin microcracks [46, 47].

A potential limitation of this study is the uneven distribution of pre-existing microcracks among the groups. Pre-preparation micro-CT images were evaluated only for calcifications to exclude related teeth, while microcracks were not assessed to maintain study integrity. Group allocation was performed randomly to minimize selection bias and contributors remained blinded to initial micro-CT images to prevent any potential influence on instrumentation procedures. Despite using ANCOVA to adjust for the differences, the imbalance in initial microcracks may still have influenced the final outcomes.

This study lacked the data about the storage conditions of the samples prior to collection and the details regarding the age of the individuals from whom the maxillary molars were obtained. In Haupt’s study, it was demonstrated that the percentage of segments with dentin defects varies significantly depending on the storage method [33]. Additionally, aging is known to alter the mechanical properties of dentin, such as its bending strength and flexibility. Age-related changes, like progressive dentin sclerosis, may weaken dentin’s resistance to the initiation and propagation of damage [34].

Another limitation of this study is the relatively small sample size, which may restrict the ability to detect minor differences between the groups. Additionally, the study was conducted in vitro, limiting its clinical relevance. Real-life factors such as chewing forces, periodontal ligament support, and intraoral moisture were not considered, making it uncertain whether the observed microcracks would develop into clinically significant vertical root fractures (VRFs) in actual patients. Reconstruction artifacts in micro-CT imaging could also influence the findings by either masking existing microcracks or generating false positives. Furthermore, the fact that all teeth were prepared by a single operator to standardize instrumentation forces, limits the generalizability of the results to real clinical scenarios, as variations in handling among different practitioners can affect the results. Another important limitation is that the study only evaluates the immediate effects of instrumentation. Long-term research is required to evaluate crack propagation over time. Lastly, the absence of a control group using manual instrumentation makes it difficult to compare the microcrack formation of rotary and reciprocating systems with that of hand files.

Future studies should aim for higher resolution imaging to capture even finer details of dentin microcracks, potentially improving the detection of smaller cracks missed in lower-resolution scans. Combining traditional sectioning and microscopy with the Micro-CT method could offer a comprehensive comparison between the two techniques, helping to validate findings and assess the limitations of each. Additionally, utilizing alveolar bone blocks during testing would more closely mimic in vivo conditions, providing results that are more applicable to clinical practice. Investigating how various tooth storage techniques affect dentin flexibility and fracture resistance could help clarify the role storage plays in microcrack formation during root canal preparation.

## Conclusion

Preparation of the mesiobuccal root canals of maxillary molars using the One Curve, VDW.ROTATE, and EdgeOne Fire systems resulted in the formation of dentin microcracks. Although the EdgeOne Fire system showed a slightly lower increase in microcracks, the differences in microcrack formation between the systems were not statistically significant.

## Data Availability

The data used during the current study are available from the corresponding author on reasonable request.

## Abbreviations

NiTi: Nickel titanium
CT: Computed tomography
